# Implementation and maintenance of patient navigation programs linking primary care with community-based health and social services: a scoping literature review

**DOI:** 10.1186/s12913-017-2046-1

**Published:** 2017-02-06

**Authors:** Ruta K. Valaitis, Nancy Carter, Annie Lam, Jennifer Nicholl, Janice Feather, Laura Cleghorn

**Affiliations:** McMaster University, School of Nursing, Hamilton, ON Canada

**Keywords:** Primary care, Primary healthcare, Community, Social services, Social care, Systems navigation, Patient navigation, Care coordination, Implementation, Scoping literature review

## Abstract

**Background:**

Since the early 90s, patient navigation programs were introduced in the United States to address inequitable access to cancer care. Programs have since expanded internationally and in scope. The goals of patient navigation programs are to: a) link patients and families to primary care services, specialist care, and community-based health and social services (CBHSS); b) provide more holistic patient-centred care; and, c) identify and resolve patient barriers to care. This paper fills a gap in knowledge to reveal what is known about motivators and factors influencing implementation and maintenance of patient navigation programs in primary care that link patients to CBHSS. It also reports on outcomes from these studies to help identify gaps in research that can inform future studies.

**Methods:**

This scoping literature review involved: i) electronic database searches; ii) a web site search; iii) a search of reference lists from literature reviews; and, iv) author follow up. It included papers from Canada, the United States, the United Kingdom, Australia, New Zealand, and/or Western Europe published between January 1990 and June 2013 if they discussed navigators or navigation programs in primary care settings that linked patients to CBHSS.

**Results:**

Of 34 papers, most originated in the United States (*n* = 29) while the remainder were from the United Kingdom, Canada and Australia. Motivators for initiating navigation programs were to: a) improve delivery of health and social care services; b) support and manage specific health needs or specific population needs, and; c) improve quality of life and wellbeing of patients. Eleven factors were found to influence implementation and maintenance of these patient navigation programs. These factors closely aligned with the Diffusion of Innovation in Service Organizations model, thus providing a theoretical foundation to support them. Various positive outcomes were reported for patients, providers and navigators, as well as the health and social care system, although they need to be considered with caution since the majority of studies were descriptive.

**Conclusions:**

This study contributes new knowledge that can inform the initiation and maintenance of primary care patient navigation programs that link patients with CBHSS. It also provides directions for future research.

## Background

Since volunteer patient navigators were introduced in the early 90s in the US to address inequitable access to care for cancer patients [1, 2], navigation programs have expanded to meet the needs of diverse populations in various settings. Navigation programs have developed internationally to address barriers to accessing health and social care for underserved populations in cancer care [3–6]. Recently, the model has been adapted for high-risk patients, such as older adults with multiple complex conditions, and to address complex health and social needs of younger populations in primary care [7–9]. In all settings, the structure and purpose of patient navigation programs vary considerably in terms of patient population, disease-focus, program design, and implementation. Despite this variability, the broad goal of navigation programs is to link patients and families to primary care services, specialist care, and community-based health and social services (CBHSS) to provide holistic patient-centred care. While there is emerging literature about patient navigation in tertiary cancer care [1, 2, 10], a fulsome understanding of the purpose, design, and implementation of navigation programs in primary care settings is now possible. This paper reports on a scoping literature review which fills a reported gap in knowledge about: motivators for implementation; factors influencing implementation and maintenance of programs; and, outcomes of navigation programs in primary care settings [11]. It must be noted, however, that the evidence of the effectiveness of navigation programs is currently limited and our aim was not to evaluate the effectiveness of these programs. A forthcoming paper will address system navigation delivery models, and roles and functions within primary care.

Given the disease-focused, episodic, and acute care orientation of health care systems, organizational and logistical challenges abound for patients, health care providers, and families navigating systems [12]. There is often a lack of care continuity and comprehensiveness, as well as limited consideration of the broader socio-economic and environmental determinants of health that have profound impacts on patients’ access to and experiences of health care [12, 13]. Health service delivery reforms have put more focus on primary care transformation, where primary care networks are promoted as the ‘hub’ of care coordination [12]. Consequently, patient navigation has emerged within primary care as a means to: a) facilitate patients’ transitions between care providers and organizations; b) create efficiencies in care integration and coordination among multiple providers and organizations, and; c) ensure that a patient’s individualized health and social needs are adequately met.

Little is known about processes that facilitate or impede implementation of navigation programs in primary care settings. This paper considers key facets of program design as articulated in the literature, with particular attention to factors related to implementation and maintenance of navigation programs. The intent is to assist managers, health care providers, and policy makers with navigation program implementation, areas to consider for outcome evaluation, as well as to identify directions for future research.

Implementation theories provide guidance on factors which can potentially influence the design and delivery of complex interventions [14, 15]. Particular emphasis is placed on the importance of understanding practical issues of implementation, such as the development and piloting of the intervention, and the value of attending to these issues in addition to evaluating program outcomes [16, 17]. Comprehensive frameworks based on these theories outline key constructs to aid in understanding attributes and processes of implementation, so that this knowledge can be shared and disseminated for uptake in other contexts. Most importantly, as Nilsen [18] points out: “Poor theoretical underpinning makes it difficult to understand and explain how and why implementation succeeds or fails, thus restraining opportunities to identify factors that predict the likelihood of implementation success and develop better strategies to achieve more successful implementation”. It is therefore important to promote the use of theory to understand and improve the process of implementation.

We have chosen the model of Diffusion of Innovations in Service Organizations (DoISO model) [14] to help us consider the theory underpinning factors that influence the implementation of patient navigation programs. The DoISO model was developed based on a meta-narrative systematic review on “a novel set of behaviours, routines, and ways of working that are directed at improving health outcomes, administrative efficiency, cost effectiveness, or users’ experience and that are implemented by planned coordinated actions” (p. 582). This work resulted in a conceptual model which was tested and validated. Its main components include: a) the innovation, b) adoption by individuals c) assimilation by the system, d) diffusion and dissemination, e) system antecedents for innovation, f) system readiness for innovation, g) the outer context: interorganizational networks and collaboration, h) implementation and routinization, and i) linkage among components of the model.

## Methods

The purpose of this scoping literature review was to reveal what is known about implementation and maintenance of patient navigation programs in primary care settings and their associated outcomes. We focused on primary care navigation that reached beyond tertiary health care to link patients and or their families to CBHSS, such as housing and employment. Our paper answers the following research questions:

1. What are motivators for implementing navigation programs in primary care?
2. What are barriers and facilitators (factors) in implementing and sustaining navigation programs?
3. What are outcomes of navigation programs?

Primary care was defined as “the first level of contact of individuals, the family and community with the national health system bringing health care as close as possible to where people live and work, and constitutes the first element of a continuing health care process” [19]. There is no commonly accepted definition of patient (or system) navigation, therefore, we refer to patient navigation as an individual or a team engaging in specific activities including:

- Facilitating access to health-related programs and services for patients/families and caregivers
- Promoting and facilitating continuity of care
- Identifying and removing barriers to care
- Effective and efficient use of the health care system for both patients/families, caregivers and practitioners

The navigation role could be taken on by a health care professional trained for this role, or a non-professional (lay person) trained to perform activities related to system navigation or a team of individuals.

### Overview and search strategy

We followed established methods for a scoping literature review as described by Arksey & O’Malley [20] and built on by Levac and colleagues [21]. One purpose of a scoping literature is to describe research in a particular field in detail for dissemination to policy makers, practitioners, and consumers. It does not include evaluation of methodological quality of studies, but provides a broad overview of research methods that dominate the topic. A scoping review draws on all types of studies regardless of design.

The search strategy involved four activities including: i) an electronic database search; ii) a web site search; iii) a search of reference lists from reviews on the topic; and, iv) email communication withan author. Databases were searched for literature published between 1990 and June 2013. These dates were chosen to capture papers published after United States cancer navigation programs were first introduced. We included papers that reported on navigators or navigation programs initiated in primary care settings in Canada, United States, United Kingdom, Australia, New Zealand, and/or Western Europe.

We used MeSH headings and free text key words applicable to two areas of interest: navigation and primary care. Terms were combined with Boolean operators ‘AND’ and ‘OR’. The research team worked with two librarians to develop a search strategy. The librarians were provided with papers from Dohan [6], Ferrante [22], Natalie-Perriera [23], Parker [24] and Sofaer [11] to assist with determining key words from primary studies highly relevant for our study. CINAHL, MEDLINE (PubMed), EMBASE, PsychInfo and CCTR databases were searched using the search terms shown in Table 1. A general internet search using Google and Google Scholar was also conducted using the marked (**) keywords in Table 2. We also contacted one author (Hendren) and retrieved two additional papers [25, 26]. Finally, relevant papers were selected from bibliographies of these nine review articles, to identify papers focused on navigators or navigation in the community.

**Table 1:** Keywords for Electronic Database Search

Preceding descriptor of navigator	Client OR patient OR community OR system OR care
Navigator	Navigat* OR Coordin*OR Facilitat* OR Network*; Care coordinator; Case manager Nurse Navigator; Patient navigator; System navigator; Personal health navigator; Promotoras; Community matron; Lay Navigator; Guided Care
Primary care	Family health team OR Group health OR Family practice OR Aboriginal Health Centre OR Community health centre OR Family health organization OR Primary care networks OR Family Health Organization OR Solo Practice Physician OR Group Practice OR CLSCs/Centre local de services communautaires OR local community service centre OR nurse practitioner led clinics OR Outpost nursing station OR (see below) Primary health care General practice General practitioners Primary care nursing Physicians/Family Patient centered care Community health services** Primary care Primary medical care Primary health care Primary healthcare Primary health service General practice** General practise** General practitioner Guided care Family practice** Family practise** Family practitioner Family health (for family health team) Family medicine Family physician Family doctor PCP** (primary care physician) Medical home
Associated Groups/Outcomes	Priority populations, social determinants of health, equity, asset-based, target populations, access

Keywords in the electronic database search marked "*" denotes keywords used in the Google and Google Scholar search

Keywords in the electronic database search marked "**" denotes a placeholder or wildcard for truncated keyword, for example, navigat* would retrieve navigation and navigator

**Table 2:** Inclusion/exclusion criteria for scoping review papers

Inclusion criteria
Published in English
Published between 1990 to June 2013
Countries of origin of study: Canada, United States, United Kingdom, Australia, New Zealand, and/or Western Europe (may have involved multiple countries, but must include at least one of those listed)
Must include the following:
• Navigator or navigation process
• Navigation role by professional or lay navigators
• Primary care setting
• Navigation that involves the community (beyond the health care system)
Was a published or unpublished primary study, descriptive paper, report, literature review using any type of method
Exclusion criteria
Published in language other than English
Published before 1990
Countries of origin of study other than Canada, United States, United Kingdom, Australia, New Zealand, and/or Western Europe
If navigation was a secondary outcome
Article did not describe in detail the extent of community navigation
Article did not address navigator or navigation process
Article did not include a navigation role by professional or paraprofessional
Article did not take place in a primary care setting
Article is an editorial, commentary or book review

### Inclusion and exclusion criteria and review process

Papers retrieved from the electronic database search had four levels of review/screening using the inclusion/exclusion criteria (Table 2). EndNote and Distiller SR were used to manage retrieved electronic papers and record reviewers’ decisions. Team members collaborated with librarians to refine the search and train for each level of review for relevancy for inclusion. In the first level, titles and abstracts of papers retrieved from the library database search were independently screened by all authors. Reviewers included papers when they were in doubt about their relevance or when there was insufficient information to make a decision. Papers assessed as relevant for inclusion by at least one team member progressed to the next level of review to maximize the number of possibly relevant papers. The next level of screening consisted of a full text review of each paper by at least two authors. When there was a disagreement at this stage, lead authors (RKV and NC) reached consensus regarding inclusion. Due to the large number of articles included at this level, the team decided to add an additional level of screening to ensure that papers were relevant to address our research questions. The third level of screening consisted of a second full text review of included papers to ensure that descriptions of navigation went beyond the health care (illness) system to include community-based health and/or social services (i.e., housing, financial support, etc.). Finally, the team extracted data from all included papers using Distiller SR and a common data extraction form created by the team.

### Analysis

Data were coded using NVivo 10 software. The research questions provided the overall framework for the coding structure which aligned with the data extraction form. The coding structure was refined with the team over multiple face-to-face meetings. Additional meetings were held to: reorganize and finalize the coding structure and collapse results into broader categories under each research question. Attributes were determined and assigned to each paper (country, publication year, research method). Finally, motivators for implementing navigation programs (question 1) and factors influencing implementation (question 2) were considered against Greenhalgh and colleagues’ DoISO model [14]. Since this is a scoping literature review, we did not systematically evaluate the methodological rigor of each paper, but did describe the research methods used [20].

## Results and discussion

**Fig. 1:**
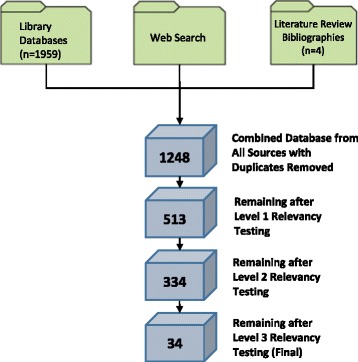
Yield from the Literature Search. This figure illustrates the main sources for the literature search including library databses, a web search, and bibliographies from relevant papers

The search yielded 1248 papers of which 34 were deemed relevant after duplicates were removed and relevancy testing was completed (see Fig. 1). We first present frequency counts of the countries of origin and research methods used in each paper, followed by motivators for implementing navigation programs, factors influencing program implementation and maintenance and reported outcomes. We have incorporated a discussion of Greenhalgh and colleagues’ DoISO model [14] to explore how the factors influencing implementation aligned with the implementation theory. We also present implications for those setting up or running navigation programs where relevant. This is followed by a summary of reported outcomes.

### Countries of origin and methods

Of 34 included papers, most originated in the United States (*n* = 29) [8, 22, 23, 25, 27–51] followed by the United Kingdom (*n* = 2) [52, 53], Canada (*n* = 2) [54, 55]; and Australia (*n* = 1) [56]. The majority were descriptive papers (*n* = 12; 34.3%) [23, 28–30, 32, 40, 43, 44, 47, 48, 50, 54]. Of the seven qualitative studies (*n* = 20%) [27, 35–37, 46, 53, 56], there were: three grounded theory studies [56], one phenomenological study [35], and one inductive qualitative study [53]. Of the seven quantitative studies (*n* = 20%) [8, 38, 41, 42, 45, 49, 52], there were three randomized controlled studies [38, 41, 42], one non-randomized controlled study [8], two cross sectional studies [45, 49] and one program evaluation [52]. There was one of each of the following: mixed methods [33], literature review [39], feasibility study [31], pilot study [22] and a retrospective, longitudinal chart analysis [34]. Three papers had unstated methods [25, 51, 55]. There were no cost-effectiveness studies.

### Motivators for the development of navigation programs

Three major motivators were identified for initiating navigation programs including the need to: a) improve the delivery of health and social care services; b) support and manage specific health needs or specific population needs, and; c) improve quality of life and wellbeing of patients. Some papers identified multiple motivators. Table 3 lists themes and sub-themes of motivators for the implementation of navigation programs.

**Table 3:** Summary of themes and sub-themes of motivators for the implementation of navigation programs

Themes: motivation for navigation programs	Sub-themes
1. To improve health and social care services delivery (*n* = 21 papers)	• Addressing access to care [31, 35, 38, 43, 45, 48, 49, 51, 53–55, 59] • Addressing health disparities experienced by high-risk groups or vulnerable populations [29, 31, 37, 43, 48, 60] • Improving care coordination among services and sectors [45–47, 53, 55] • Informing programming through disease surveillance to [30, 53, 54] • Reducing hospital and emergency room admissions [52, 53] • Ensuring access to holistic primary care services [51, 53] • Increasing screening and follow up for abnormal results [31] • Improving efficiency in resource use [8] • Providing supports to family physicians to address complex mental health and addictions issues [55]
2. To support and manage particular health needs or specific population needs (*n* = 18 papers)	• Supporting management of patients with: o Mental health and/or addictions [33, 38, 51, 54, 56] o Chronic disease including multiple co-morbidities [27, 41, 42, 45] o Cancer [31, 36, 48] o HIV [44, 48] o Primary immunodeficiency disease [40] • Managing primary health care needs of: o Parents and their children [51] o Pregnant women dealing with domestic violence [50, 51] o Physical health problems to prevent long-term disability [38]
3. To improve the quality of life and well being of patients (*n* = 7 papers)	• Improving: o General health and wellbeing of families [38, 42, 48] o Quality of life [8, 40] o Self-management skills and empowering patients [48, 53]

Regardless of motivators for navigation programs, the DoISO model maintains that an innovation should show “relative advantage” [14] (p.594), such as increased effectiveness or cost-effectiveness, for easy adoption. Cost effectiveness was not identified explicitly as a motivator in these papers, although it was an outcome explored by some authors [29, 43, 45, 52] that was mostly measured in relation to reduction in emergency room visits, institutionalization, and hospitalization. Providers initiating patient navigation programs should consider explicitly identifying motivators for them so that benefits may be easily visible to adopters to support implementation.

### Factors influencing successful implementation and maintenance of navigation programs

No papers focused specifically on implementation of navigator roles or navigation programs. However, in our analysis, descriptions of navigation programs pointed to many factors during phases of program development, implementation, and evaluation. Factors are organized based on these phases. The next section provides an overview of each factor with its elements (Table 4). Each factor is examined in relation to the DoISO model where relevant.

**Table 4:** Factors and their elements influencing implementation and maintenance of navigation programs

Factors	Elements describing each factor
1. Patient Characteristics:	• Complexity of clients/patients • Need to address clients/patients basic needs (e.g., shelter) first • Caregivers of clients/patients are patients themselves • Geographic restrictions (e.g., access to services in rural communities) • Language barriers • Respect for cultural values
2. Effective Recruitment and Training of Navigators	• Recruitment of lay navigators supported by word of mouth • Maintenance of ongoing training to support: o Growth and development of navigators, o Role transitions o Problem solving for complex cases o Collaboration and mutual support among navigators o Orientation to the needs of the population being served by navigators
3. Role Clarity	• Clear boundaries set for navigators (particularly lay navigators) in their role o Clarifying role boundaries with patients/clients as well as physicians • Valuing role clarification • Management of anxiety when taking on new navigation role to build confidence
4. Effective and Clear Operational Processes	• Careful development of planning processes • Development of policies and procedures to support program activities • Establishment of documentation mechanisms such as clinical intake forms • Use of consensus decision-making approaches • Provision of clinical supervision and steering committee oversight • Regular communication between agencies for planning purposes • Mechanisms to address scheduling and referral challenges
5. Adequate Human, Financial, and Tangible Resources including Technological Resources	Provision for: • Human resources o Dedicated, committed, engaged and adequately trained clinical staff o External availability of experts such as attorneys • Financial resources o Secured external funding • Tangible resources o Appropriate space for navigator and navigation work • Technological Resources o Internet resources to locate resources and support complex cases o Electronic health records (EHR) to support documentation of evidence based care plans, patient assessments o EHR to support access to community resources, coordinate transitions, and promote self-management o Email or phones to support communication with physicians • Adequate time to support transitional care and provide comprehensive care to a large caseload.
6. Strong Inter and Intra Organizational Relationships/Partnerships:	• Encouraging commitment from all professionals involved • Establishment of self-governing team environment in the practice (supports role development) • Development of strong relationships with community agencies by: o Development of a community charter o Establishment of a community-based steering committee o Development of communication strategies with partner agencies o Mechanisms to address inter-organizational issues with power differentials and other tensions between agencies
7. Lack of Available Services in a Community	• Addressing the problem of “navigation to nowhere” (Inadequate or non-existent local services)
8. Effective Communication between Providers	• Encouragement of consistent attendance at regular meetings by staff (monthly) • Sharing of updates related to patient/client progress (through EHR) regularly • Involvement of physicians in meetings regularly • Communication between all care providers
9. Program Uptake and Buy In by End Users of the Program	• Selling/getting buy in to the navigation program with consumers • Use of diverse strategies for recruitment to programs o Recruitment strategies are not successful with all population groups (i.e., outreach,) need to be tailored • Addressing potential stigma in getting participation in mental health navigation programs
10. Valuing of navigators	• Valuing navigators by providing them with opportunities to be recognized and heard
11. Evaluation of navigation programs	• Evaluation of navigation programs: o Developing evaluation plan with team for ongoing evaluation o Considering community-based participatory research approaches o Focusing on program related processes (degree to which mission/goals are met) o Considering using pre-identified indicators o Addressing potential problems with lack of access to data, monitoring health status changes over time attribution of outcomes to navigation interventions

#### Factor 1: Patient characteristics

Eight papers raised issues related to patients’ characteristics including needs, interests, or values as having an impact on navigation program implementation and uptake. One paper recommended identifying a clear vision of patients’ needs and determining priorities to support implementation [22]. Some papers reported that clients’ basic needs, such as affordable housing, should be addressed before navigation programs would work [30, 31, 54]. Challenges arose for nurses in a Guided Care Program for complex seniors as caregivers were often patients themselves and this likely hampered attendance at workshops and support groups offered by the program [42]. In another paper, clients’ language barriers challenged uptake of a navigation program [43]. Community Health Worker (CHW) navigators who provided community referrals, health education, and screened for adherence to medications for their working poor clients found that ensuring respect for cultural values was important for program success [35].

The DoISO model posits that simpler innovations are more easily adopted than those that are complex [14]. Many patients in navigation programs – end users of the innovation – are vulnerable. Consequently, they require more complex interventions to address their concerns. Thus, client characteristics will likely directly influence the complexity of the program innovation needed to effectively serve them. Those introducing navigation programs should carefully consider client characteristics which can influence successful implementation as it will likely need to be a complex intervention given the purposes and populations served by navigation programs. Further, Greenhalgh and colleagues [14] note compatibility (with adopters’ values, norms, and needs) and reinvention (ability to adapt, refine or modify the innovation to meet adopters’ needs) as factors influencing implementation. Therefore, programs should be designed and tailored for delivery to be compatible and consistent with patient and or population characteristics.

#### Factor 2: Effective recruitment and training of navigators

Navigator training was important in program implementation. Ongoing training to support: growth and development [37], role transitions [41], problem solving for complex cases [48], and collaboration and mutual support among navigators [36] helped maintain navigation programs. Challenges included the absence of orientation to population needs being served by navigators [51], and ambivalence of navigators in relation to attending training [37]. Also, inadequate staff training was problematic for program sustainability [27]. One paper touched on methods of lay navigator recruitment which was supported by word of mouth through health and community services provider networks rather than job postings [36]. Recommended topics for training case managers, navigators, and outreach workers included motivational interviewing, the stages of change model, strength-based approaches to working with clients [34], and preparation for management of emotional needs [36].

In the DoISO model, understanding the innovation well – i.e., what it does and how including role clarity – is important as a prerequisite for adoption of an innovation in the pre-adoption stage [14]. It is not surprising then, that navigator training was a factor raised in a number of successful navigation programs to ensure provider understanding and uptake. Providing support for navigators to help them understand how the role might affect them personally is encouraged. The DoISO model also suggests that relevant training, such as timely on-the-job training; the availability of a help desk; and high quality training materials to support uptake and assimilation in practice can support implementation. Furthermore, the use of team-based training for more complex interventions was shown to be effective [14].

#### Factor 3: Role clarity

Five papers specifically addressed issues related to role clarity of navigators. Spiro and colleagues highlighted the value in setting clear boundaries for CHWs that they are not to provide any clinical advice [30]. Layne et al., [43] also highlighted the value of role clarification in relation to the importance of patients’ roles as partners in their care. Nurses working in a Guided Care Program identified a degree of anxiety about expectations in their new multi-faceted role and a lack of confidence in some topic areas [41]. These concerns waned over time with clinical experience and nurses felt that they had mastered the expanded role.

Maintaining boundaries with patients with complex health or social needs was problematic for some social worker navigators and the extended time required to provide social work roles and functions created tension between patient navigators and some physicians [22]. Retkin and colleagues [47] reported on a program to support cancer patients and survivors with legal issues and engaged attorneys to support the patient navigators who felt poorly prepared to deal with certain topics (e.g., workplace and social security issues).

The DolSO model points out that interventions can have a ‘hard core’; “the irreducible elements of the intervention” and ‘soft periphery’ which allows for adaptations to the intervention in the structure of the organization [14]. This is referred to as having ‘fuzzy boundaries’ which is viewed as supporting system readiness for reinvention of an innovation. Lack of role clarity could be viewed as the program lacking a ‘hard core’. This emerged as a significant challenge for some programs both with professionals as well as lay navigators. A subsequent paper will explore the concept of roles that navigators play in more depth.

#### Factor 4: Effective and clear operational processes

Eight papers touched on operational processes, which were facilitators or barriers to implementing navigation programs. Facilitators included careful development of planning processes [50] and support from steering committee members to review cases and service data [46, 54]. Other facilitators included policies and procedures that were well articulated [50] and supported program activities, such as: problem solving around cases, decision-making around clarifying boundaries of work and referrals (when to refer on), safety procedures for home visits, communication strategies between partners to conduct planning activities, and documentation [30, 42, 50, 54]. Decision-making processes that carefully thought through models of service to meet community needs and consensus decision making was also valued [54]. Finally, clinical supervision and steering committee oversight in a program for patients with mental health and addictions enabled the ability to track changes in the scope of service in the program [54]. The largest operational process barrier was scheduling and availability of appointments and loss to follow up [43, 49, 50].

The review highlighted the value of policies and procedures to support navigation program activities such as documentation and referral, clinical oversight, and decision-making. These elements are supported to some extent by the DoISO model, which supports the need for formal decision-making processes for “planned and sustained efforts at implementation” [14]. It also speaks to the need for assimilation by the system through changes in structures and ways of working. Greenhalgh and colleagues stated that if knowledge required to use the innovation “can be codified and transferred from one context to another”, then it is more likely to be adopted [14]. The development of policies and procedures appears to support the transfer of such knowledge and should be encouraged.

#### Factor 5: Adequate human, financial, and tangible resources and time

Having adequate human, financial and tangible resources were important factors influencing implementation and maintenance of navigation programs. Supports included having: secured external funding [50]; dedication of committed and engaged clinical staff [50, 55]; and external availability of content experts, such as attorneys to address legal concerns of patients [47]. Some papers reported the use of technology to facilitate navigators or navigation programs. Three papers [8, 30, 41] mentioned the use of electronic health records (EHRs) to create individualized evidence-based care and record patient assessments. EHRs and information systems were also used to facilitate access to community resources [32], coordinate transitions in care, educate caregivers, and promote self-management [42].

Having adequate time was a reported resource challenge. Boyd et al., reported that it took time to integrate the Guided Care Nurse into the clinic work flow due to physician skepticism with the role [8]. Another challenge was the time it took to: provide comprehensive care; address challenging patient problems such as the management of transitional care; and, address large caseloads [41].

Other resource challenges included physicians concerns about establishing a funding pool [22] and maintaining funding for navigators [27, 55]. Many recommended ongoing funding for navigation programs [27, 34, 55] noting that CHW activities are not reimbursable [30]. Others recommended leveraging partnerships with agencies to obtain funding support [30] or working with community partners for support [55].

Space also needs to be taken into consideration. One study found that adequacy of space influenced the amount of time spent with clients and subsequent contribution to collaborative care [22]. Proximity to other organizations can enhance the delivery of comprehensive services [32].

The DoISO model supports the need for dedicated ongoing funding as well as time and human resources to support implementation and routinization of innovations [14]. Both the scoping review and the model speak to the positive influence of having well-trained staff to support implementation. The model focuses on early and widespread involvement of staff to promote successful implementation. This was not identified in the scoping review, however, it should be strongly considered. Although not noted in the model, this review highlighted the important challenges of space in the implementation of navigation programs. This is not surprising given the environment in primary care [57] where space dedicated to support special programming is often at a premium. The need for adequate time to address transitional and comprehensive care to a large caseload, points to the issue of complexity of navigation programs and populations they serve which was discussed earlier. Technology supports were raised in a number of our papers in our review. The DoISO model [14] identifies structural determinants of organizations that support innovativeness, which includes slack resources (resources available “beyond minimum requirements to maintain operations” p. 605) and technical capacity. Given the strong evidence related to the essential need for adequate resources, people implementing navigation programs need to ensure there is dedicated time for the implementation of navigation programs, the navigator role itself, as well as technical and space resources.

#### Factor 6: Strong inter and intra organizational relationships/partnerships

Both inter and intra-organizational relationships were identified as facilitators by many authors. There was value in having strong relationships with community agencies to reach target populations and offer comprehensive services [30, 46, 50, 55, 56]. One paper focused on the importance of commitment from all professionals involved [50]. Useful strategies for maintaining strong relationships included: the development of a community charter [55], the establishment of a community-based steering committee [54, 55], building community partnerships to increase target population reach [30], and the development of communication strategies with partner agencies to sustain relationships and foster community cohesion [46]. Role development was fostered by having a self-governing team environment in the practice [28].

Challenges were also reported in relation to inter and intra organizational relationships. Community-based steering committee challenges included difficulty getting buy-in for navigation programs addressing addictions and mental health despite having organizations sign a charter [54]. In another study, Ferrante and colleagues [22] reported poor understanding of the navigator role and a lack of intraorganizational collaboration between the health care team and the navigator who serviced patients with complex needs.

The DoISO model also argues that boundary spanners (people in the organization who have strong social ties to others inside and outside of the organization) are important for dissemination of the innovation [14]. This is a critical implementation given the nature of navigation programs and the need to work with services to refer and link patients to supports. It is important to build strong inter and intra organizational relationships with community agencies and all professionals involved on teams to be successful.

#### Factor 7: Lack of available services in a community

A significant challenge identified in a rural community was ‘navigation to nowhere’. Anderson and colleagues struggled with the lack of community services to help address social issues such as affordable housing, poverty, and transportation [54, 55]. Further research is warranted to explore how to overcome the problem of ‘navigation to nowhere’. This challenge moves beyond the implementation of navigation programs to highlight inequities in availability of health and social services, which is beyond the scope of this paper. However, it emphasizes the importance of assessing the community before implementing a navigation program that may not be feasible in communities with limited available health and social services. It also highlights the challenge for program developers to explore resources outside of their home communities accessible through telehealth or other technologies [58, 59].

#### Factor 8: Effective communication between providers

A factor critical to navigation program success was effective communication supported through regular meetings [41, 46, 48, 50, 54]. Primary care team members also benefited from general face-to-face communication and information sharing through EHRs [30]. Physician engagement in meetings was integral in a program supported by community health liaison staff who connected patients to community health resources and attempted to increase staffs’ perceived value of the role and learn from them [46].

In the DoISO model, intraorganizational communication demands effective communication across organizational structural boundaries to increase the chance of routinization of the innovation [14]. The model argues for the importance of communicating accurate and timely feedback about the impact of implementation. Papers in our study focused more on communication with primary care team members and partners in relation to patient care rather than the implementation process itself. It may be that this occurred but simply was not reported. The model also speaks to having formal dissemination programs to support successful implementation focusing on communication around the innovation. This was not reported in the literature but should be considered in future program implementation.

#### Factor 9: Program uptake and buy-in by patients

A few papers discussed challenges related to strategies used to ‘sell’ the program. They described programs that promoted outreach navigation services tied to primary care centres. In one paper, student navigators (ranging from first year undergraduates to senior medical students) helped the homeless population navigate the healthcare system by running health fairs in soup kitchens [32]. McCloskey et al. [37] used weight training groups to recruit males to their community-based outreach program for Hispanics with diabetes. In a US study, patients with risk conditions (e.g., homebound or requiring mental health or social services) were not able or willing to pay for navigation support leading to poor uptake [22]. Program uptake can be successful if the purpose of the program is based on community needs [50].

Greenhaugh’s model argues that the meaning of the innovation in the eyes of the adopter has a powerful influence on uptake. Although the DoISO model does not directly discuss strategies to increase uptake, it notes that the innovation should have a clear relative advantage. A lengthy process of negotiation to convince adopters of the programs’ merit may be needed where the meaning of the innovation is debated [14]. This may not be possible to do with patients in navigation programs, but may be easier with service providers [14]. Ultimately, navigation program implementation requires diverse and tailored strategies to address needs and concerns of different populations to sell its benefits and advantages through evidence, discussion, pilot testing, and observation to enhance uptake.

#### Factor 10: Valuing of navigators

One study found that valuing navigators by providing them with opportunities to be recognized was a facilitator: placing equal value on CHW navigator input with other team members was another crucial implementation facilitator [30]. This was not raised in other papers or in the DoISO model. However, evidence on implementation of nurse practitioners roles in health care shows the importance of demonstrating the value of a new role, as well as valuing the person in that role for successful implementation [60, 61].

#### Factor 11: Evaluation of navigation programs

Evaluation of navigation programs was reported by some authors as helping with program development and maintenance. One author reported using multiple strategies such as: team development of an evaluation plan including the CHW; the use of internal evaluators; and, application of a community-based participatory research process [30]. Some evaluations focused on program-related processes (tracking daily work and routine reporting), as well as the degree to which they were meeting the program’s mission and meeting goals [30, 33, 41, 50]. Giddens et al’s. Guided Care Model used pre-identified indicators, the results of which were shared with navigators. This resulted in opportunities for education and problem-solving towards program improvements [41]. Sustainability and generalizability were identified as key measures of success by one author [55]. Barriers to evaluation were also reported including: lack of access to appropriate data [50]; difficulty monitoring health status change in patients with chronic diseases over time [38]; and, difficulty attributing outcomes to navigation interventions due to the complex nature of the role [30]. A number of papers reported the need for continuous evaluation at regular intervals [29, 46], measurement of changes in community health indicators [30, 46], and commitment to collecting, analyzing and disseminating results to the community [55].

The DoISO model addressed the importance of evaluation in implementation of innovations [14]. Having a formal planned dissemination program including monitoring progress with milestones and a rigorous evaluation is suggested. A formal evaluation phase may be the norm when an innovation is complex and involves the whole organization in which structural or organizational changes are needed. Evaluating implementation of a navigation role in a primary care setting may at first seem a simple task, but given the challenges seen in the implementation of new roles in primary care, it may not be as simple as it first appears [62, 63].

### Outcomes of navigation programs

A number of outcomes of navigation programs for patients, providers and navigators, as well as the larger health and social care system were reported. Table 5 outlines reported outcomes in detail with accompanying citations. Ten papers reported on outcomes for patients that were related to improvements in general health and wellness such as, reduced unmet needs, improved mental health, and reduced co-morbidities. This was followed by seven papers which described improved self-efficacy, self-management or empowerment. In addition, five papers reported increased patient satisfaction regarding services for themselves or their children. One paper captured a negative experience in a cancer patient navigation program delivered by community health workers (CHW) working in a variety of settings including primary care [36]. Female breast cancer patients were uncomfortable receiving services from a male navigator and in addition, there was lack of care continuity and poor navigator follow up.

**Table 5:** Reported outcomes of patient navigation programs

Outcomes	Citations
**Patient and caregiver outcomes**	
*Positive Outcomes*	
Improvements in general health and wellness	
• Reduced unmet needs	[38, 44]
• Improved quality of life	[23, 44]
• Improved mental health	[33]
• Improved activities of daily living	[33]
• Reduced co-morbidities	[23]
• Improved understanding of patients’ health conditions and their management	[35, 43]
• Decreased worries, concerns and stress	[23, 44]
• Reduced caregiver strain or depressive symptoms	[41, 42]
• Improved biomarkers for chronic disease (e.g., HbA1c; fewer HIV clients with a detectable viral load	[29, 44]
Improved self-efficacy, self-management or empowerment	[23, 30, 38, 46, 51].
Increased patient satisfaction regarding services for themselves or their children	[23, 30, 38, 46, 51].
Increased access to care:	
• Care overall (i.e., increase in number of patients seen)	[22]
• A primary care medical home	[23, 51]
• Timely primary care	[38];
• Medications	[43]
• More access to culturally appropriate care	[23, 37, 49]
• Specialty or sub-specialty care (for children; for AIDS/HIV patients)	[44, 51]
Better follow up and uptake of screening:	
• Reduced missed medical appointments	[43]
• For legal counsel	[47]
• Increased patient encounters and communication with primary care	[8, 23, 33, 44, 46]
• More mammography or cancer screening according to guidelines	[23, 31]
Financial, employment, and health claims addressed	
• Increased employment and reduced financial stresses	[33]
• Reduced numbers of mental health patients who applied for disability benefits, with significantly higher behavioural health claims	[33]
• Proportion of patients suffering from mental illness who become insured	[23]
• Patients connected to legal services reported positive impacts on finances and compliance with medical appointments and treatment	[47]
• More affordable services for working poor	[35]
*Neutral or negative outcomes*	
Discomfort with male navigators for female breast cancer care, lack of care continuity and poor navigator follow up	[36]
No differences in employment, hours worked or earnings	[38]
**Provider outcomes**	
Satisfaction with navigation programs	[30, 41, 46]
Increased communication among primary care providers and community services or providers	[8, 46, 54, 55]
Increased knowledge and skills	[47, 48]
Increased trust between	
• Navigators and physicians	[41]
• Patients and their attorneys	[47]
Improved care coordination	[47, 55]
Navigators empowered in their community advocacy role and were promoted in their positions	[30, 37]
**Health system outcomes**	
Reduction in emergency room and/or hospital use	[28, 29, 43, 45]
Prevention of premature institutionalization	[52]

Increased access to care in general was another commonly reported patient outcome. In one case, there was no difference seen in access to primary care or specialists for a predominantly older (over 75 year old) population [45]. In addition, particular aspects of access were improved such as access to: a primary care medical home, primary care as soon as it was needed; specialty or sub-specialty care for children and referrals; culturally appropriate care and medications. Better follow up care and uptake of cancer screening including use of guidelines in screening was also found. Patient encounters and communication with primary care were increased with navigation programs such as increased visits, improved communication, more reviews, check-ins and/or goal setting conducted and links made to other providers. Financial and employment concerns as well as and health claims were also being addressed with outcomes such as: increased employment, reduced financial stresses, improved insurance coverage. One paper reported no differences in employment, hours worked or earnings [38]. Two randomized controlled trials focused on caregivers of older patients with multiple chronic conditions applied the Guided Care Program with nursing support. In the trial led by Foret Giddens et al., [41] caregivers felt less strain. This was supported by Wolf and colleagues [42] who found modest benefits in reducing depressive symptoms as well as caregiver strain among caregivers with their navigation program.

Provider outcomes were reported in nine studies including feelings of satisfaction with navigation programs as well as increased communication among primary care providers and community services and among providers. Findings also showed that there were increased knowledge and skills in topics such as legal issues and chronic conditions; increased trust between navigators and physicians as well as patients and their attorneys. Others found improved care coordination and follow up. Positive outcomes were also reported specifically for navigators. Promotores were empowered in their community advocacy role and some were promoted into supervisory roles [37]. CHWs who were professionally trained in their home countries had an opportunity to redevelop as professionals to work in the US [30]. Few papers reported on health system outcomes, which included a reduction in emergency room and/or hospital use [28, 29, 43, 45] and the prevention of premature institutionalization for older adults [52].

Overall, there were many reported benefits resulting from navigation programs. This provides evidence that such programs warrant consideration as a means of improving health, increasing access to care (particularly for more vulnerable populations with complex needs), improving navigator and provider experiences and care delivery, and reducing use of costly hospital services. Only one paper reported minor negative results of a navigation program involving trained CHWs. Given the preponderance of benefits of navigation programs, it is imperative that we understand factors influencing their implementation to enhance future program development and implementation.

## Discussion

A strength of this study was the rigor and breadth of the literature search and the stringent methods used to conduct the scoping literature review. One challenge that we encountered in our review was difficulty in identifying relevant papers for inclusion. This was due to the vague or limited descriptions of patient navigation interventions, including the extent of navigation. There were many instances where it was not clear if or how links were made to CBHSS. Given that linking patients in primary care to CBHSS was the focus of our review, when the description was unclear in relation to these links, we chose to exclude the paper. As a result, we may have excluded some papers in which primary care patient navigation programs did link to CBHSS. We strongly encourage authors to use the TIDiER checklist [64] in describing their interventions to aid in syntheses of all relevant literature and comparisons of results.

A large number of included papers were descriptive. Further, three papers did not name a research method indicating poor methodological rigor. The number of descriptive reports was somewhat surprising given the size of the yield and the fact that primary care researchers often focus on quality improvement initiatives [65, 66]. Our review also included some controlled quantitative studies as well as in-depth qualitative studies. Despite this range in the strength of methods used, we found evidence of a number of factors across papers that can support successful implementation and maintenance of primary care patient navigation programs that link patients to CBHSS. They also aligned with the DoISO model [14, 67]. There were no studies, however, that specifically focused on evaluating implementation identified in this review. This gap could be addressed to further validate our factors influencing implementation.

Given the aims of scoping literature reviews, and the fact that the majority of papers were of a descriptive nature, it is not possible to draw any conclusions related to the effectiveness of patient navigation programs in this study, nor was this our aim. Our results, however, did indicate that there were various positive outcomes reported suggesting that patient navigation programs are of value in primary care. A systematic literature review of primary care-based patient navigation programs is warranted as a next step to answer questions related to program effectiveness. The National Cancer Institute’s Patient Navigation Research Program [68, 69] comprehensively explored outcomes of navigation programs in cancer care and could be a useful model to guide this work. Furthermore, we support future effectiveness-implementation hybrid trials that can measure both implementation and outcomes of patient navigation programs [70].

## Conclusions

In conclusion, this scoping literature review revealed that the implementation and maintenance of navigation programs in primary care requires attention to a number of complex factors. This is not surprising since navigation programs are typically focused on meeting the needs of complex vulnerable populations or those with multiple chronic health and social conditions. Implementation factors that emerged from the literature were supported by Greenhalgh and colleagues’ DoISO model [14], providing empirical support for the model as well as theoretical support for the factors found to influence implementation and maintenance of patient navigation programs in primary care that linked to CBHSS. This review therefore can be useful for those planning to realize similar programs in primary care.

It is important to note that some elements of the DoISO model were not found in our review, such as: a) intentional strategies to spread the innovation; b) the influence of political directives; and c) the role of opinion leaders or champions, although the lack of physician support was found to be a barrier in one paper [8]. Future research and practice could benefit from further investigation of these factors that can influence successful implementation. In addition, research that examines which implementation factors are most important and in which contexts is warranted. For example, are some factors more important than others based on the population served or the health or social issue being addressed? Comparative longitudinal case studies could be helpful to better understand this question. Consideration of the potential barriers and facilitators (factors) in implementing and maintaining navigation programs as well as strategies for proactively addressing them as identified in this study can help mitigate implementation and uptake problems.

## Abbreviations

CBHSS: Community-based health and social services
CHW: Community health worker
DoISO: Diffusion of Innovations in Service Organizations Model
